# Paramagnetic NMR to study iron sulfur proteins: ^13^C detected experiments illuminate the vicinity of the metal center

**DOI:** 10.1007/s10858-023-00425-4

**Published:** 2023-10-18

**Authors:** Leonardo Querci, Deborah Grifagni, Inês B. Trindade, José Malanho Silva, Ricardo O. Louro, Francesca Cantini, Mario Piccioli

**Affiliations:** 1 Magnetic Resonance Center and Department of Chemistry, University of Florence, Via L. Sacconi 6, 50019 Sesto Fiorentino, Italy; 2 Instituto de Tecnologia Química e Biológica António Xavier (ITQB-NOVA), Universidade Nova de Lisboa, Av. da República (EAN), 2780-157 Oeiras, Portugal; 3 Division of Biology and Biological Engineering, California Institute of Technology, CA 91125 Pasadena, USA

**Keywords:** Paramagnetic NMR, ^13^C NMR, Iron-Sulfur proteins, Optimized ^13^C experiments, Transverse relaxation, NEET proteins

## Abstract

The robustness of NMR coherence transfer in proximity of a paramagnetic center depends on the relaxation properties of the nuclei involved. In the case of Iron-Sulfur Proteins, different pulse schemes or different parameter sets often provide complementary results. Tailored versions of HCACO and CACO experiments significantly increase the number of observed C^α^/C’ connectivities in highly paramagnetic systems, by recovering many resonances that were lost due to paramagnetic relaxation. Optimized ^13^C direct detected experiments can significantly extend the available assignments, improving the overall knowledge of these systems. The different relaxation properties of C^α^ and C’ nuclei are exploited in CACO vs COCA experiments and the complementarity of the two experiments is used to obtain structural information. The two [Fe_2_S_2_]^+^ clusters containing NEET protein CISD3 and the one [Fe_4_S_4_]^2+^ cluster containing HiPIP protein PioC have been taken as model systems. We show that tailored experiments contribute to decrease the blind sphere around the cluster, to extend resonance assignment of cluster bound cysteine residues and to retrieve details on the topology of the iron-bound ligand residues.

**Supplementary Information:**

The online version contains supplementary material available at 10.1007/s10858-023-00425-4.

## Introduction

The direct detection of ^13^C NMR is routinely used in biomolecular NMR to complement ^1^H detected experiments and to obtain information that cannot be obtained via ^1^H detection (Bermel et al. 2006a; Felli and Pierattelli 2022). Usually, the gain in sensitivity is given by the polarization transfer from ^1^H to ^13^C spins, which largely overcomes the signal loss due to the ^1^H transverse relaxation during the INEPT transfer and, in many applications, provides better results than ^13^C-start experiments (Shimba et al. 2004; Bermel et al. 2009; Richter et al. 2010; Pontoriero et al. 2020; Vögeli et al. 2005; Pritchard and Hansen 2019). However, in paramagnetic metalloproteins, nuclear relaxation rates depend on the square of the gyromagnetic ratio (γ^2^) of the nucleus investigated, on the r^−6^ dependence from metal-to-nucleus distance and, for the first coordination sphere, on the amount of unpaired electron spin density delocalized from the metal ion(s) to the nuclear spins (Miao et al. 2022; Pell et al. 2019; Trindade et al. 2022; Bertini et al. 2017). Therefore, the choice of the most efficient experiment is not trivial: complementary results are often obtained when the same experiment is recorded with different pulse schemes (Arnesano et al. 2003; Gelis et al. 2003; Invernici et al. 2020; Ciofi-Baffoni et al. 2014) and “non-systematic” assignment strategies, tailored according to the relaxation properties of specific spin systems, need to be defined (Trindade et al. 2021a).

In protein NMR, backbone C^α^-C’ connectivities are very useful for sequence-specific and site-specific assignments, because of the correlation between chemical shift values and amino acid type (Mao et al. 2014). Many experiments have been designed to identify homonuclear C^α^-C’ connectivities (Bertini et al. 2005a; Lee et al. 2005; Machonkin et al. 2002; Kostic et al. 2002; Bax 2011), which can also be used to address macromolecular dynamics and interactomics (Stenstrom et al. 2022; Mori et al. 2010; Ferrage et al. 2006). In paramagnetic systems, relaxation losses easily make a ^13^C-start experiment comparable and eventually more sensitive than a corresponding ^1^H-start experiment (Pontoriero et al. 2020; Balayssac et al. 2006). The intrinsic asymmetry of CACO experiments can also be exploited: CACO is based on a single coherence transfer step from the excited nucleus to the observed nucleus, therefore transferring magnetization from C^α^ to C’ or vice versa, in the COCA experiment, would call into the scene different relaxation rates. As a consequence, complementary information are obtained from C^α^ to C’ and C’ to C^α^ transfer pathways (Machonkin et al. 2002; Bertini et al. 2005b), contributing to characterize the first coordination sphere of a metal center. In the absence of magnetic anisotropies of the metal ions, when no structural information can be obtained from chemical shifts (Pintacuda et al. 2007; Zhu et al. 2023; Wu et al. 2022; Herath et al. 2021; Muntener et al. 2022; Parker et al. 2020), the proximity of the metal center can be monitored only by mapping the paramagnetic relaxation of nuclear spins nearby and each individual spin system has a different behaviour, depending on its topology vis-à-vis the metal center (Trindade et al. 2021a).

Within this frame, we considered the behaviour of two different paramagnetic metalloproteins: CISD3 and PioC, both containing iron-sulfur clusters. At variance with other metalloproteins, in which the electron relaxation is driven by the coordination number of the metal ion (Spronk et al. 2018; Ravera et al. 2021; Beniamino et al. 2022; Trindade et al. 2021b), the electronic correlation times in iron-sulfur proteins are determined by the magnetic coupling among the iron ions. Therefore, different FeS clusters affect their protein environments at different extent (Blondin and Girerd 1990; Banci et al. 2018, 1990; close to the molecular mechanism understanding 2018; Bennett et al. 2022; Azam et al. 2020; Gervason et al. 2019; Stegmaier et al. 2019; Mulliez et al. 1999; Trindade et al. 2023). CISD3 (CDGSH Iron Sulfur Domain-3) is a 93 aminoacids protein containing two [Fe_2_S_2_]^+^ clusters (Karmi et al. 2018; Tamir et al. 2015). Each cluster is coordinated by a 3-Cys, 1-His biding motif, embedded into a highly conserved domain, called CDGSH domain. The protein is a monomer and the two CDGSH domains are similar but not equivalent. Both clusters are in the reduced [Fe_2_S_2_]^+^ form, formally containing one Fe^3+^ and one Fe^2+^ ion (Golinelli-Cohen et al. 2016; Gee et al. 2021; Camponeschi et al. 2022). The crystallographic structure of the protein is available only for the H75C/H113C mutant (Lipper et al. 2018). We used CISD3 because an extended assignment of the protein has been recently obtained from a combination of standard and tailored ^1^H and ^13^C detected experiments (Silva et al. 2023). The system is particularly challenging because [Fe_2_S_2_]^+^ clusters are efficient relaxing agents (Camponeschi et al. 2021; Cai et al. 2017; Pochapsky et al. 2001; Valer et al. 2022) and paramagnetism affects about 60% of residues in CISD3 (Grifagni et al. 2023), preventing the identification of the NMR signals of the cluster-bound residues and the resolution of the NMR solution structure. PioC, a High Potential Iron Protein (HiPIP) from *Rhodopseudomonas palustris* TIE-1 (Bird et al. 2014), is a very small protein (54 amino acids) that contains a single [Fe_4_S_4_]^2+^ cluster. The electron relaxation times of iron ions in [Fe_4_S_4_]^2+^ clusters are shorter than the [Fe_2_S_2_]^+^ case, for this reason the detection of C^α^/C’ connectivities of iron-bound cysteine residues can be attempted and a high-quality NMR structure has been obtained (Trindade et al. 2021c) (PDB ID: 6XYV).

Together, these two iron-sulfur proteins will assess the performances of CACO experiments in systems characterized by different relaxation properties. On the one hand, we will show how the optimization and the combination of ^1^H and ^13^C start experiments contribute to improve the resonance assignment in highly paramagnetic systems. On the other hand, when signal losses due to paramagnetism are less severe and a complete resonance assignment can be obtained, the structural dependence of paramagnetic relaxation enhancements can be exploited in many intriguing ways.

## Results and discussion

### Tailored HCACO experiments contribute to resonance assignment: the case of CISD3

CISD3 is a small protein that contains two [Fe_2_S_2_]^+^ clusters. Triple resonance NMR experiments recorded using routine parameter sets, permit the identification of only ca 40% of the protein residues, consistent with a detectability threshold of about 9 Å from each iron ion of the cluster (Camponeschi et al. 2021). Our interest here is to discuss the complementarity of various CACO experiments (Bertini et al. 2005a; Bermel et al. 2003, 2005a). In particular, the comparison of ^1^H vs ^13^C start CACO experiments promise to reveal unique information on the metal to nucleus distances of the spin systems that are affected by the paramagnetic center.

**Fig. 1:**
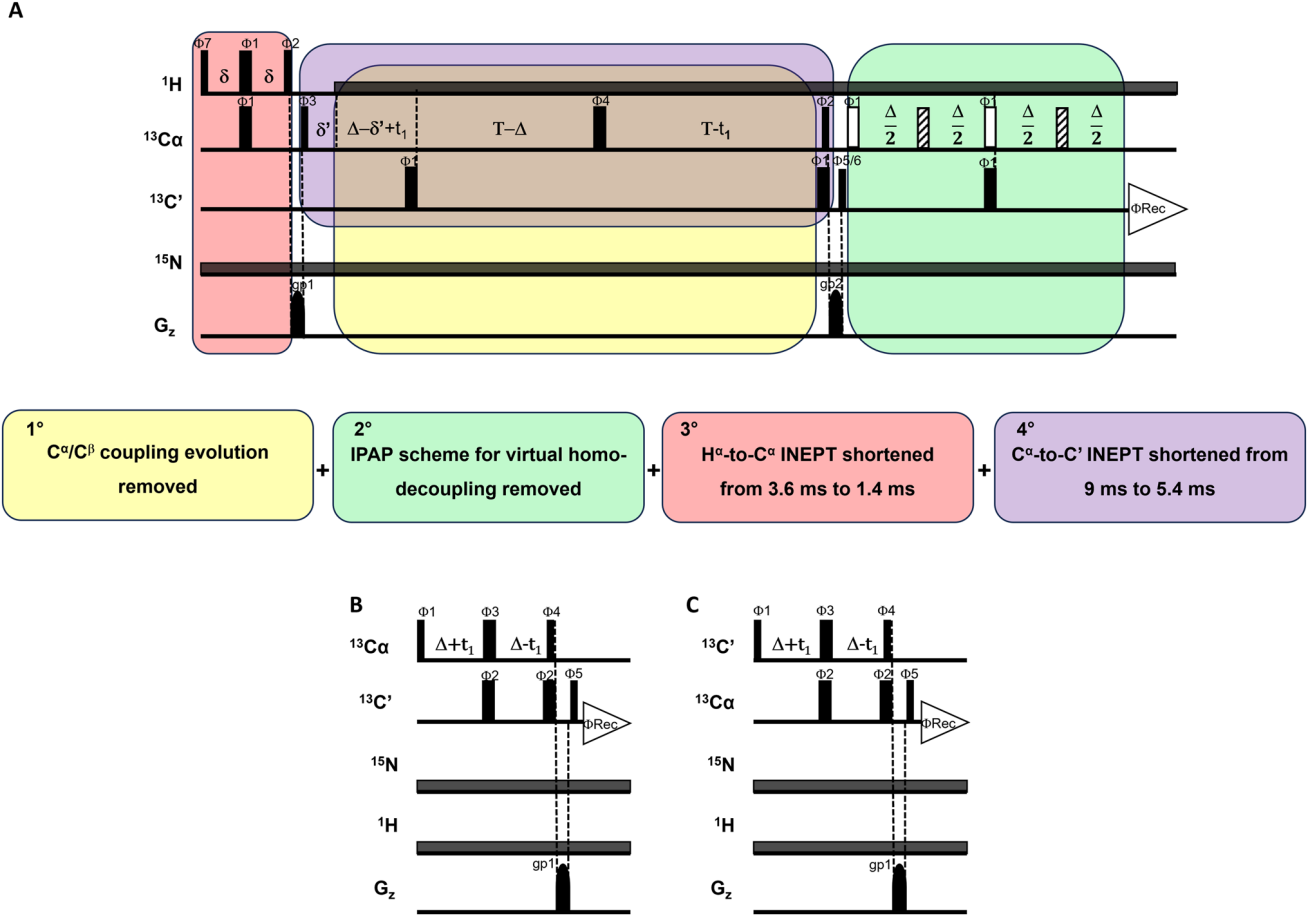
**A** Standard HCACO experiment. White and dashed 180° pulses indicate IPAP scheme for virtual homo-decoupling of C^α^/C coherence. The colored panels highlight parts of the pulse scheme that have been modified during the optimization process. The delays utilized in the routine HCACO experiment are the following δ = 1.8 ms, δ’ = 1.1 ms, Δ = 4.5 ms, Τ = 14.2 ms. The first modification, highlighted in yellow, eliminates the C^α^/C^β^ coupling evolution period. T is set equal to Δ = 4.5 ms, the two 180° C^α^ and C’ pulses are applied together. The next modification, highlighted in green, removes the IPAP scheme for virtual homo-decoupling. The entire block is removed and acquisition begins immediately after the last 90 °C’ pulse. In the subsequent step, highlighted in red, the H^α^-to-C^α^ INEPT delay δ was shortened from 1.8 ms to 0.7 ms. The last modification, highlighted in purple, shortens the C^α^-to-C’ INEPT delay ∆ from 4.5 ms to 2.7 ms. The phase cycle, maintained throughout the modifcations, is: Φ1 = x; Φ2 = y; Φ3 = x, − x; Φ4 = x, x, x, x, x, x, x, x, y, y, y, y, y, y, y, y; Φ5 = x, x, x, x, − x, − x, − x, − x; Φ6 = − y, − y, − y, − y, y, y, y, y; Φ7 = x, x, − x, − x; ΦRec = x, − x, − x, x, − x, x, x, − x, − x, x, x, − x, x, − x, − x, x. **B** The CACO-AP and **C** COCA-AP pulse schemes implemented with a C^α^-to-C' INEPT delay equal to Δ = 2.7 ms. The phase cycle for the two experiments is the following: Φ1 = x; Φ2 = y; Φ3 = x, − x; Φ4 = x, x, x, x, y, y, y,y; Φ5 = x, x, − x, − x; ΦRec = x, − x, − x, x, − x, x, x, − x. Gradient strenght was gp1 = 30%, gp2 = 50%. PFG gradients used in all the pulse sequences had a sine bell shape and duration of 1 ms. The period set for the dissipation of circulating currents was 200 μs

**Fig. 2:**
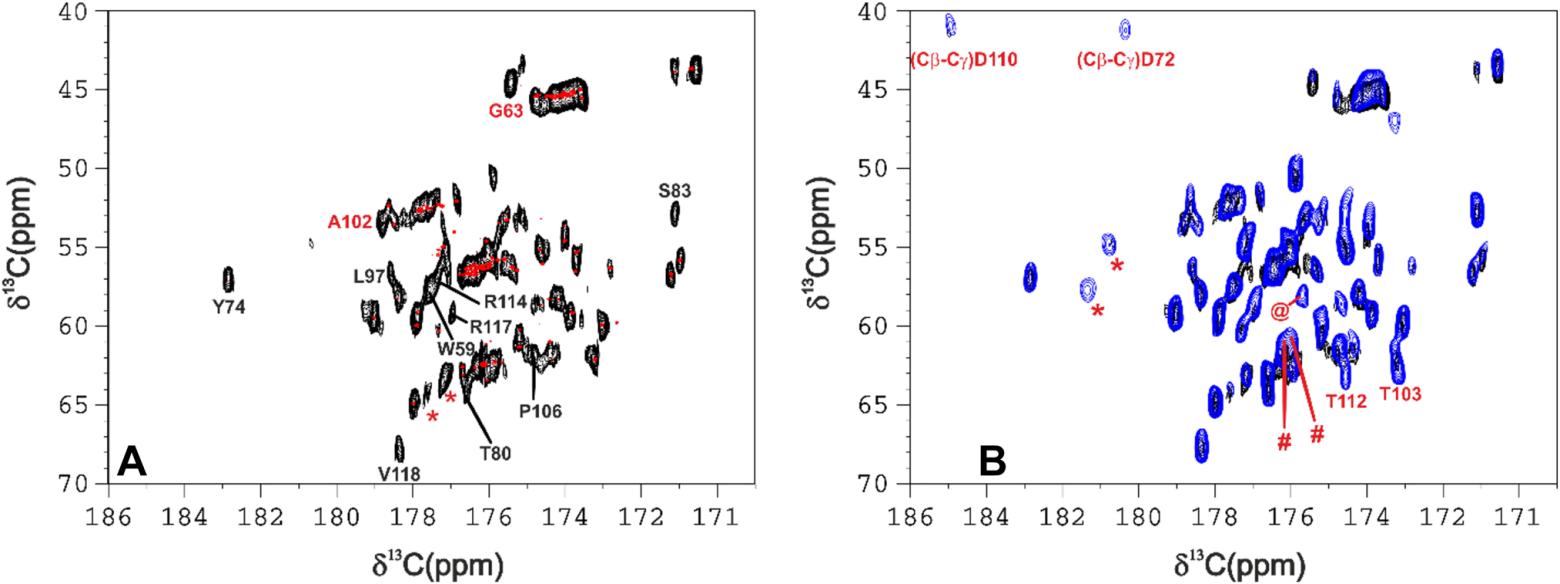
175 MHz, 298 K NMR spectra of reduced CISD3. **A** HCACO spectra obtained using the sequence reported in Fig. 1A. The spectrum shown in red (standard dataset) has been obtained with the following parameters: acquisition (t_2max_) and recycle delays 92 ms and 1 s; δ = 1.8 ms, δ’ = 1.1 ms, ∆ = 4.5 ms, T = 14.2 ms. 32 scans each FID were recorded over a 1024 × 660 data point matrix. Total experimental time was 6.5 h. The spectrum shown in black (paramagnetic tailored dataset) has been obtained with acquisition (t_2max_) and recycle delays 46 ms and 400 ms; δ  = 1.4 ms, δ’ = 1.1 ms, T  = ∆ = 2.7 ms. 1024 scans each FID were recorded over a 512 × 50 data point matrix. Total experimental time was 6.5 h. Assignment reported in black shows residues that could be sequence specifically assigned with a set of paramagnetic tailored experiments^60^, labels in red indicate peaks that were identified, and some of them assigned, in this experiment. **B** HCACO (black) vs CACO-AP (blue). The spectrum shown in black is the same reported in panel A, the spectrum shown in blue has been obtained with the pulse sequence reported in Fig. 1B, with acquisition (t_2max_) and recycle delays 24.5 ms and 300 ms. The C^α^-to-C’ INEPT period was set to ∆ = 2.7 ms. 1024 scans each FID were recorded over a 512 × 70 data point matrix. Total experimental time was 6.5 h. Labels show peaks that were observed only in the CACO-AP and discussed throughout the text

First, we compared two HCACO experiments recorded with the same pulse sequence (Fig. 1) but using two different parameter sets. One experiment was recorded with a conventional parameter set, optimized for a small/medium sized protein, the other was recorded by optimizing all delays for the detection of fast relaxing signals and for minimizing signal losses. While 43 signals are observed in the “routine” version of the HCACO and then sequence specifically assigned, thirteen additional signals appeared in the “paramagnetic” spectrum. Out of them, nine signals have been sequence specifically assigned (black-labelled peaks in Fig. 2A) using a combination of standard and tailored triple resonance NMR experiments (Grifagni et al. 2023). These residues are at H^α^-Fe distances in the range 7–8.7 Å (except for Thr 80 and Ser 83, both in the linker region between the two CDGSH domains, where some conformational rearrangements in solution might take place), indicating that the blind sphere of the optimized HCACO experiment has decreased. The four peaks identified but not yet assigned in the tailored HCACO are also expected to be within the same H^α^-Fe distance range, this significantly restricts the number of possible candidates for their assignment. Moreover, all nuclear spins within this distance range from the paramagnetic center should experience paramagnetic relaxation but no paramagnetic shift (Banci et al. 2018; Camponeschi et al. 2019), therefore chemical shift values can also be used for signal assignment. In the tailored CACO spectrum of Fig. 2A, the signal at 175.4/44.5 ppm can be safely attributed to a glycine residue. There are three unassigned glycine residues in CISD3, all located in proximity to an iron ion. Only **Gly 63** has an H^α^ more than 6 Å apart from the closest iron ion. This is the only distance compatible with the observed HCACO peak, and therefore we assign this signal to Gly 63. Likewise, the peak at 178.7/53.5 ppm is assigned because these chemical shifts are characteristic of alanine residues and the only alanine residue not yet assigned is **Ala 102**, which has the H^α^ at 6.1 Å from the iron ion. We are then left with two signals at relatively downfield C^α^ values (labelled with red asterisks in Fig. 2A), whose C^α^/C’ shifts might be consistent with valine, phenilalanine or leucine. The only candidates for the assignment are Val 61, Phe 76 and Leu 82, which have H^α^-Fe distances of 5.7 Å, 7.0 Å and 7.9 Å, respectively. Among them, we might speculate that Val 61 is probably too close to the iron ion to be observable and, therefore, **Phe 76** and **Leu 82** are the most likely candidates for the two unassigned peaks of Fig. 2A.

### ^*1*^*H start vs *^*13*^*C start experiments*

To compare the features of ^13^C- vs ^1^H-excitation, we performed a CACO-AP experiment, in which both the initial H^α^-to-C^α^ INEPT and the final C^α^-to-C’ IPAP are removed, and signal detected as a C’ antiphase doublet, phased in dispersion mode (Bertini et al. 2005a). The pulse sequence for this experiment is reported in Fig. 1B. As expected, for signals that are not affected by the paramagnetic center, the sensitivity of the CACO-AP experiment is lower than the HCACO. Nevertheless, Fig. 2B shows nine additional signals in the CACO-AP, compared to the ^1^H^α^-start HCACO. These resonances have not been observed in other ^1^H start experiments (with a few exceptions discussed here below), because their scalar coupled ^1^H nuclei are too close to the metal to be observed, with estimated metal-to-proton distances shorter than 7.0 Å. Protonless experiments, in which fast relaxing protons are never involved into the coherence transfer pathway (Balayssac et al. 2006), provide the best coherence pathways to circumvent signal losses due to paramagnetic relaxation enhancement. As outlined previously in the case of tailored HCACO, signals identification within a given experiment is associated to a well-defined distance range between electron and nuclear spins. When a structure is available, the predicted distance range and the chemical shifts information allow us to propose some assignments. Among signals exclusively observed in the CACO-AP experiment (Fig. 2B), two of them fall in the peculiar CACO region of threonine residues. There are three unassigned threonine residues in CISD3: Thr 99, Thr 103 and Thr 112, all belonging to the C-term CDGSH domain and close to the C-term cluster (cluster 2, hereafter). The signal at 173.1/62.9 ppm is completely missing in the tailored HCACO, however in the CON experiment (data not shown), there is a non-assigned correlation between a C’ at 173.1 ppm and a, still unassigned, N backbone atoms at 116.6 ppm. This pattern suggests that this CACO peak belongs to a threonine residue with an H^α^ very close to the metal center but with the C’ nucleus relatively far from the iron ion, enough to allow the detection of its CON peak. The nucleus-to-iron distances of all unassigned amino acids, which are reported in Table S1**,** are well consistent with the assignment to **Thr 103**, connected with Gln 104, whose HN group has not been assigned. The other threonine peak, observed in the CACO-AP at 174.5/63.3 ppm, is barely detectable also in the HCACO experiment but it does not have any possible connectivity in the CON spectrum, indicating a different orientation of this spin system versus the cluster, compared with Thr 103. We expect for this residue larger Fe-H^α^ and shorter Fe–C’ distances than Thr 103. According to Table S1**,** this is the case of **Thr 112**. By exclusion, Thr 99 would remain unobserved, because Thr 99 has shorter metal-to-carbon distances than the other two threonine residues. The two signals at 184.9/41.1 and 180.2/41.3 ppm can be attributed to C^β^-C^γ^ connectivities of aspartate residues. According to the crystallographic structure of the H75C/H113C double mutant, both carboxylate groups of **Asp 72** and **Asp 110**, which are the only two aspartate residues of the protein, are at about 6 Å from the iron and, therefore, they might be observable in a CACO-AP experiment. Their residue specific assignment is much more tentative: both carboxylate groups are expected to form H-bonds with Ser 74 and Thr 112, respectively. At variance with Asp 72, the side chain of Asp 110 is also very close to the guanidinium moiety of Arg 105, which might explain the unusual downfield shift of aspartate C^γ^ signals and suggest the assignment proposed in Fig. 2B. There are five additional signals observed in the CACO-AP experiment that, following previous considerations, belong to residues with C^α^/C’ distances in the range 5.0/6.5 Å from the metal center, i.e. the distance range experienced by the herewith assigned threonine backbone C^α^/C’ and aspartate side chains C^β^/C^γ^ signals. Possible candidates, according to Table S1, are: Pro 46, Arg 64, Asp 72, Ser 74, Phe 76, Pro 84, Lys 101, Asp 110, which are in the proper distance range and are still unassigned. Among possible candidates we do not consider the iron-bound cysteine and histidine residues, because their C^α^ should experience considerable hyperfine shift (Querci et al. 2023). The two signals at 176.2/61.5 ppm and 175.9/60.9 ppm, labelled with **#**, are in the Pro-Phe region and possible candidates are Pro 46, Pro 84 and Phe 76. On the other hand, the signal at 175.7/57.9 ppm, labelled with **@**, is within the Arg-Lys region and, therefore, candidates are Arg 64 and Lys 101. There is no possible assignment based on chemical shifts predictions for the two peaks at 181.2/57.7 ppm and 180.7/55.0 ppm, labelled with *. Unusual C’ shift values may arise from the network of H-bonds around the two clusters: it has been shown that H-bonds are important to modulate the reduction potential of individual iron ions (Langen et al. 1992) and contribute to the delocalization of unpaired spin density from the iron ions to the backbone nitrogen atoms involved in H-bonds (Lin et al. 2009; Xia et al. 1999; Trindade et al. 2020). Unpaired electron delocalization might be partly transferred also to the C’ atom of the preceding residue. Ser 74, Asp 110 and also the two previously mentioned Arg 64 and Lys 101 might experience this effect. Even if these peaks are unassigned, they can be utilized to map intermolecular interactions. Indeed, they clearly arise from the immediate proximity to the cluster and are probably the NMR observables closest to the cluster that can be acquired.

### Optimization of the HCACO experiment: complete resonance assignment of iron bound residues in HiPIP protein PioC

**Fig. 3:**
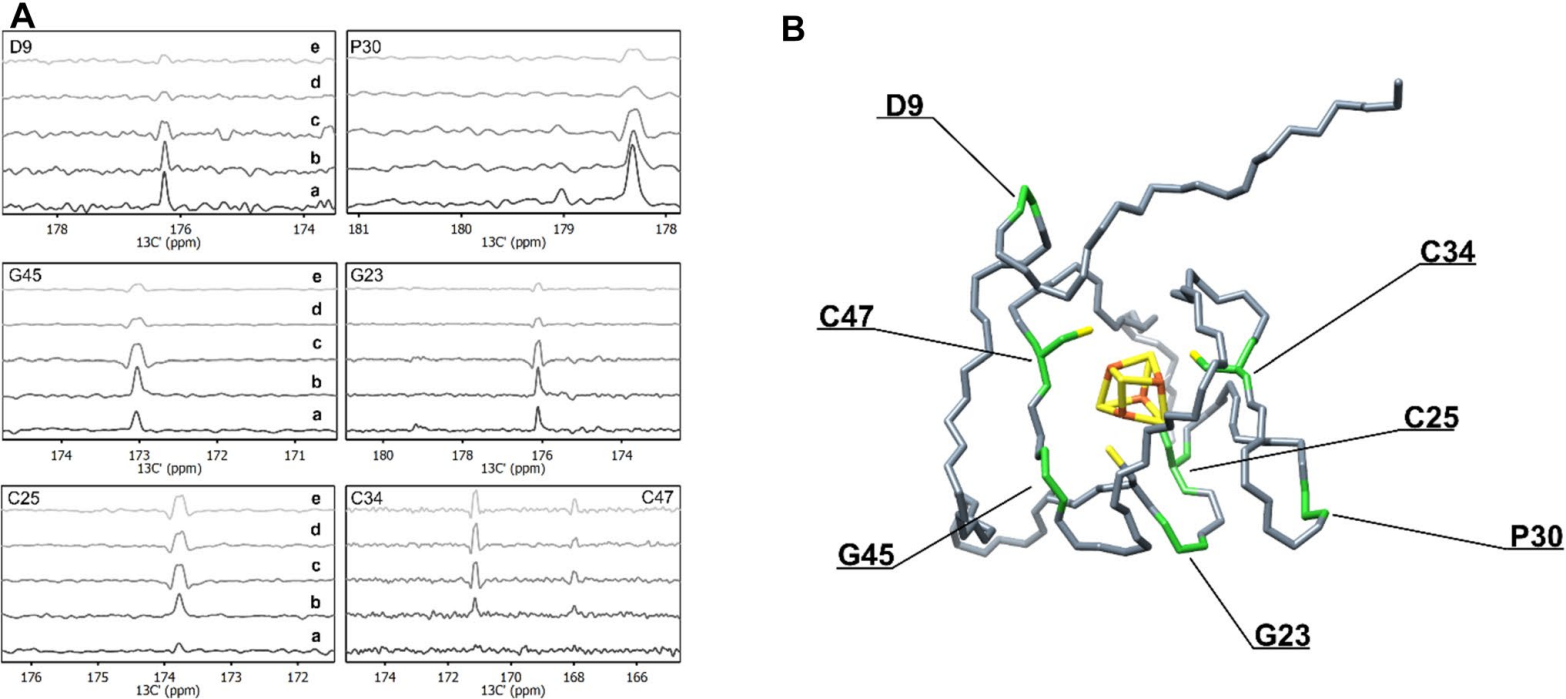
**A** Stacked rows extracted from five different HCACO experiments for seven residues of the HIPIP protein PioC. Each panel show a selected row of the HCACO experiment recorded with modified versions of the experiments, as presented in Fig. 1A and re-summarized here: a: standard pulse sequence; b: no C^α^/C^β^ coupling; c: no C^α^/C^β^ coupling & no IPAP; d: no C^α^/C^β^ coupling & no IPAP & shorter H^α^/C^α^ INEPT period; e: no C^α^/C^β^ coupling & no IPAP & shorter H^α^/C^α^ INEPT & shorter C^a^/C' INEPT evolution. **B** Lowest energy NMR conformer of PioC (PDB ID: 6XYV). Residues shown in panels A are labeled and marked in green

**Fig. 4:**
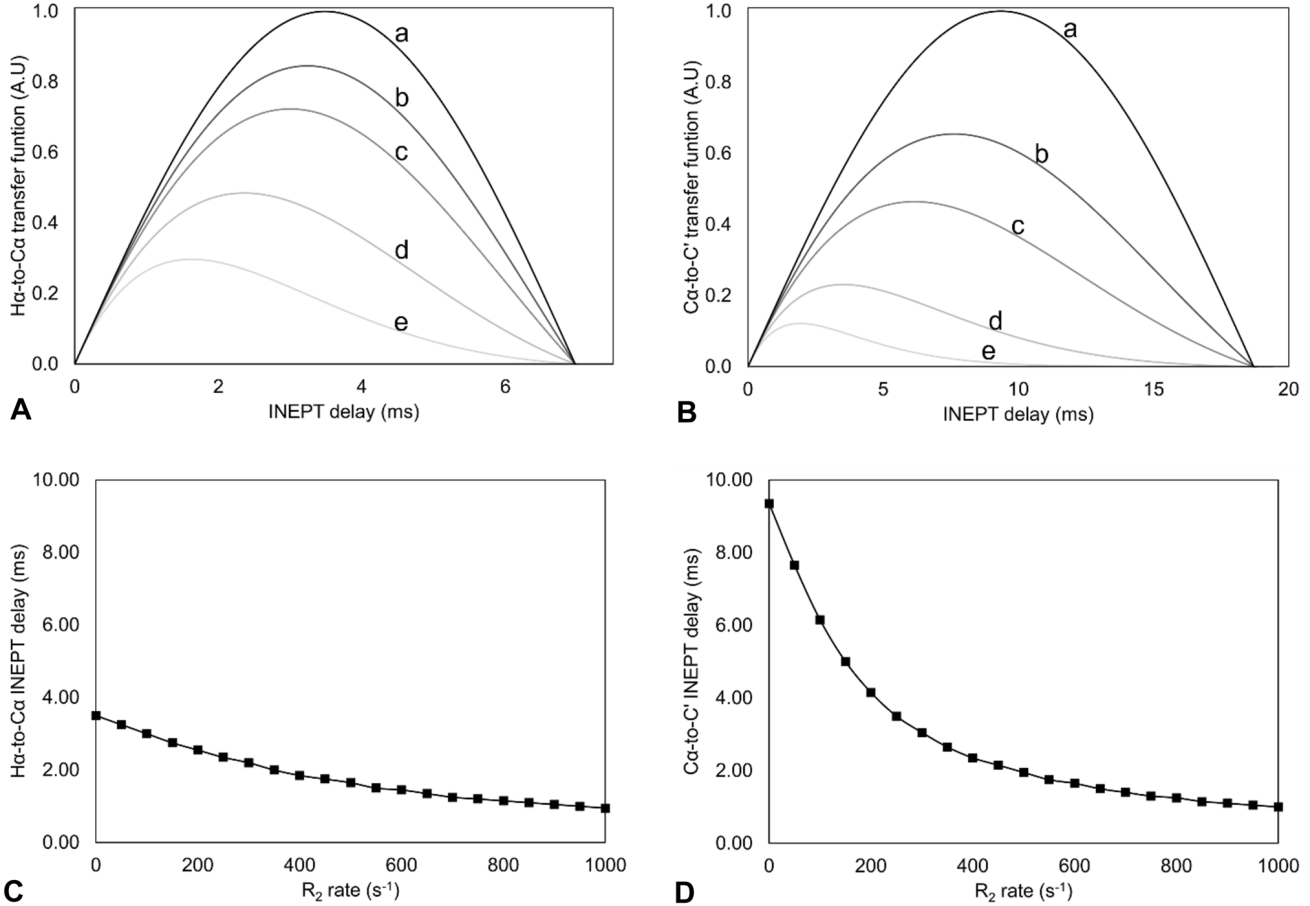
Calculated relaxation (R_2_)-weighted transfer functions for the **A** H^α^-to-C^α^ and **B** C^α^-to-C’ INEPT steps of HCACO pulse sequence, simulating H^α^ and C^α^ nuclear spins with increasing transverse relaxation rates. a: neglected R_2_ relaxation (R_2_ = 0 s^−1^) b: R_2_ = 50 s^−1^, c: R_2_ = 100 s^−1^, d: R_2_ = 250 s^−1^ and e: R_2_ = 500 s^−1^. The maximum of the transfer function decreases with increasing transverse relaxation rates. Best calculated INEPT delay for **C** H^α^-to-C^α^ and **D** C^α^-to-C’ coherence transfer at different R_2_ rates

We considered as a test system for the optimization of the HCACO experiment, the HiPIP protein PioC, containing a [Fe_4_S_4_]^2+^ cluster. At variance with CISD3, PioC is a very stable protein, its NMR assignment is almost complete and signals from residues that bind the metal center can be observed. PioC is therefore a paradigmatic case for the quantitative assessment of HCACO performances. A series of HCACO experiments, recorded with the same acquisition parameters (same number of scans, t_1_ increments, acquisition and recycle delays) was recorded at 1.2 GHz, and the peak intensities are reported for some selected peaks in Fig. 3A. The lower panels show the C^α^/C’ connectivities of Fe-bound cysteines, that are the residues most affected by paramagnetic relaxation; the middle panels report peak intensities of residues that are close to the paramagnetic center but do not belong to the first coordination sphere; the upper panels show peaks unaffected by paramagnetic relaxation. Figure 3B shows the location of these residues within the protein frame. In the routine experiment (traces a), Cys residues are missing or only barely visible, the two Gly residues are weak and signals far from the paramagnetic center are recorded with good sensitivity. The stepwise implementation of the pulse sequence is schematically shown in Fig. 1A and it allows one to analyse the contribution of each parameter to the overall effects. The removal, in the F_1_ dimension, of the constant time needed to refocus the homonuclear C^α^/C^β^ coupling (traces b), significantly contributes to revive signals affected by paramagnetic relaxation at the expenses of the sensitivity of signals not affected by paramagnetism. Similar effects are observed when the final IPAP scheme is replaced and signals detected as C’ antiphase dispersion doublets (traces c). These two modifications are essential for the detectability of cysteine signals, but they also improve the intensities of the residues affected by paramagnetic relaxation at lower extent, like the two glycine residues Gly 23 and Gly 45 (Fig. 3A middle panels): whenever a significant paramagnetic contribution to relaxation occurs, these two implementations do contribute to increase the number of observable peaks. Further improvements can be obtained by shortening the delay of the H^α^-to-C^α^ INEPT (d) and of the C^α^-to-C’ INEPT (e). The sensitivity gain in these cases is strictly dependent on the behavior of the relaxation weighted coherence transfer functions, which are reported in Fig. 4. In Fig. 3A (lower panels), we observe that, when H^α^-to-C^α^ and C^α^-to-C’ INEPT delays are decreased from their “standard” values of 3.6 ms and 9.0 ms to, respectively, 1.4 ms and 5.4 ms, signals of Fe bound Cys 25, Cys 34 and Cys 47 increase in intensity, although at different extent. On the other hand, signals of Gly 45 and Gly 23 decrease the intensity. The impact of relaxation rates on the coherence transfer affects both the efficiency of the transfer and the choice of the optimal delay. As shown in Fig. 4C and D, optimal transfer delays decrease at increasing R_2_ values and, as a consequence, at decreasing metal-to-nucleus distances. In the case of the experiments reported in Fig. 3A, delays were calibrated according to very fast nuclear relaxation, with the aim to identify signals close to the detection limit. Indeed, we observe an increase in signal intensity for C^α^/C’ of Cys residues at the expenses, in this case, of the intensity of those signals that are affected by paramagnetism at a lower extent.

**Fig. 5:**
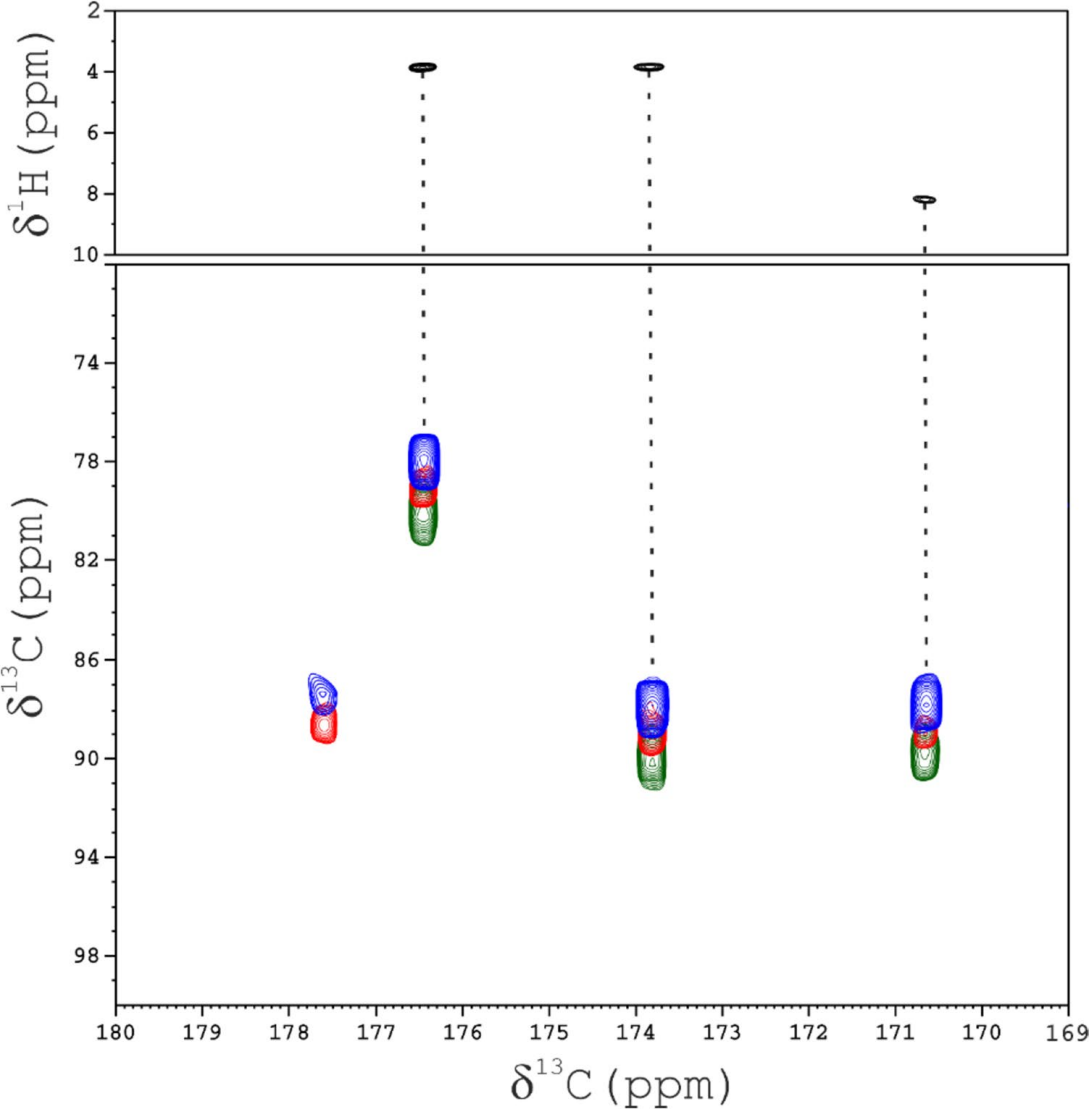
CACO spectra of PioC acquired at 175 MHz at 298 K. The C^α^ carrier of the F_1_ dimension is placed at 86 ppm, in order to excite selectively the hyperfine shifted C^α^ Cys signals. Green contours: HCACO-AP recorded with 1.4 ms as H^α^-to-C^α^ INEPT transfer; blue contours: HCACO-AP recorded with 1 ms as H^α^-to-C^α^ INEPT transfer; red contours: CACO-AP experiment. For the sake of clarity, red and blue spectra have been arbitrarily shifted in the F_1_ dimension. The top panel shows the H(CA)CO-AP version of the experiment, recorded with acquisition, recycle and H^α^-to-C^α^ INEPT transfer delay of 46 ms, 250 ms and 1.4 ms, where the H^α^/C’ connectivities are obtained

Essentially, H^α^-to-C^α^ and C^α^-to-C’ polarization transfers act here as relaxation-based filters that select signals according to transverse relaxation rates. Figure 5 shows optimized HCACO-AP and CACO-AP spectra recorded on PioC, in the spectral region where C^α^/C’ connectivities of cluster bound cysteines have been assigned. Four signals, from the four iron-bound cysteine residues are observed in the CACO-AP experiment. The optimized HCACO-AP experiment, recorded with an H^α^-to-C^α^ INEPT transfer period of 1.4 ms, gives only three signals; indicating that the H^α^_y_C^α^_z_ coherence of Cys 22 relaxes faster than the other three cysteine residues and its signal is lost prior to ^13^C^α^ t_1_ evolution. However, a further decrease of the transfer delay from 1.4 ms to 1 ms, succeeded to revive Cys 22 C^α^/C’ connectivity also in an optimized HCACO-AP experiment (blue contour plot, in Fig. 5).

The HCACO can be easily modified to evolve H^α^ instead of C^α^ during t_1_, i.e. an H(CA)CO experiments (Serber et al. 2000; Bermel et al. 2006b; Duma et al. 2003). This was particularly useful in the case of PioC, where the “missing piece” of the complete resonance assignment involve H^α^ of Cys residues. Three of the four cysteines’ H^α^ protons could be unambiguously assigned with this experiment. In particular, the H^α^ at 8.18 ppm was previously observed in a ^13^C-HSQC experiment and, because of the severe overlap of three C^α^ cysteines’ shifts, it was previously assigned to H^α^ of Cys 22. In the H(CA)CO-AP experiment (Fig. 5, upper panel), the H^α^/C’ correlation allows us to correctly assign the H^α^ at 8.18 ppm to Cys 47, leaving the H^α^ of Cys 22 as the only unassigned resonance of the first coordination sphere of the cluster.

### Mapping the geometry of cluster bound residues: CACO-AP vs COCA-AP

**Fig. 6:**
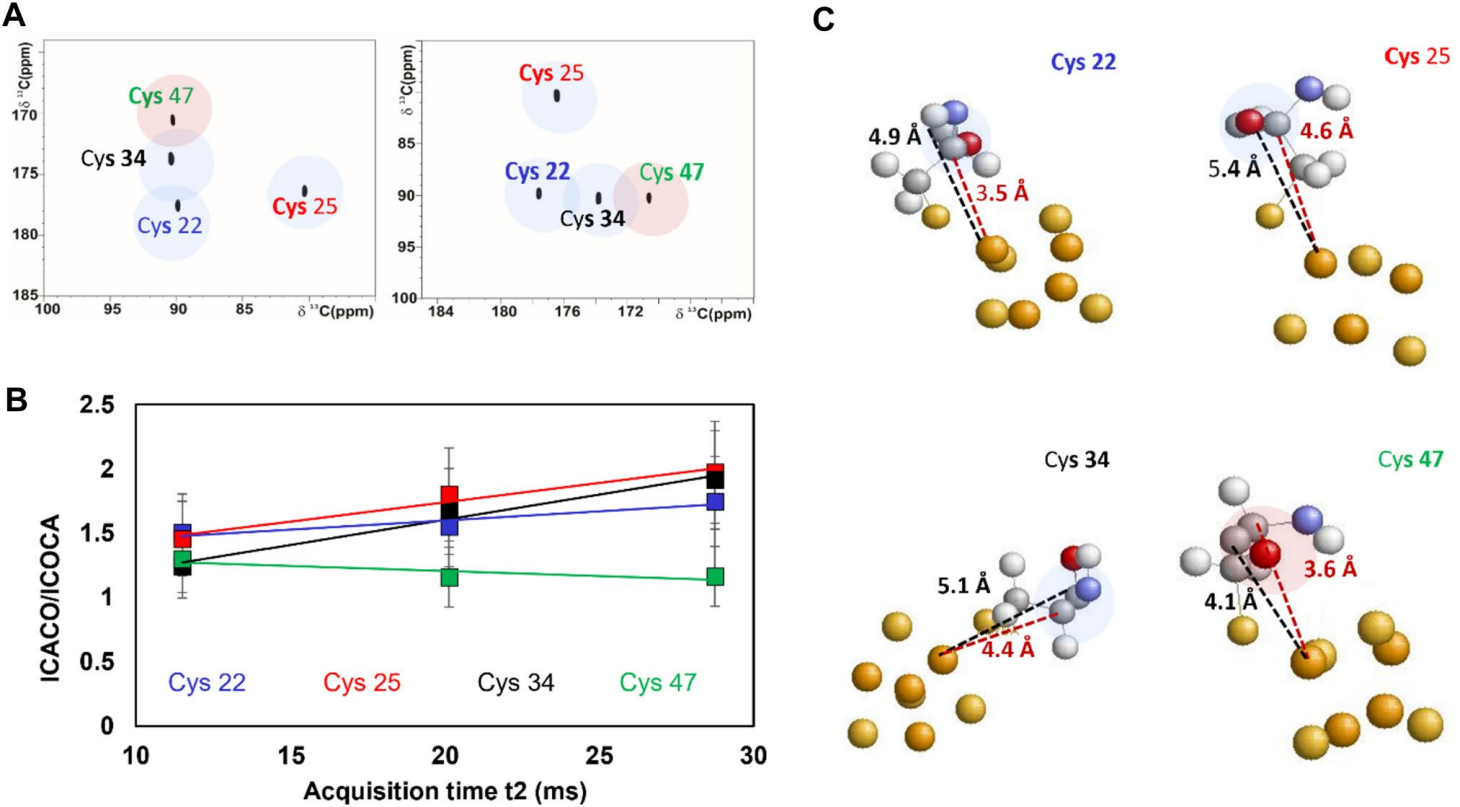
**A** COCA-AP (left-half) and CACO-AP (right-half) spectra. **B** I^CACO^/I^COCA^ peak intensity ratios related to increasing acquisition time in the direct dimension of the spectrum (t_2_). Peak intensities have been calculated by performing the experiments using a 46 ms acquisition time in the direct dimension and then considering for FT a smaller number of effective data points. **C** Molecular view of PioC’s cluster binding residues, atoms are coloured as follow: hydrogen (white), carbon (gray), oxygen (red), nitrogen (blue), iron (orange) and sulfur (yellow); different topologies of C^α^-C’ vector are presented. C’-Fe and C^α^-Fe distance are marked in black and red dashed lines, respectively

The relaxation properties of C^β^, C^α^ and C’ of iron bound cysteines depend on the orientation of backbone and side chain atoms with respect to the iron ions; different topologies of metal bound residues originate distinctive spectroscopic features. A promising tool to study this effect is the comparison of COCA-AP and CACO-AP experiments, shown in Fig. 6.

The two pulse sequences are identical and only the C^α^/C’ frequencies are swapped (Fig. 1B, C). The experiments are based on a single coherence transfer step from the excited nucleus to the observed nucleus; performing the transfer from C^α^ to C’ or viceversa would call into the scene different relaxation rates. Therefore, the relative intensity of COCA and CACO peaks depends on the different topologies of metal binding side chains. We recorded, on the [Fe_4_S_4_]^2+^ protein PioC, COCA-AP and CACO-AP under the same experimental conditions using recycle, coherence transfer and t_1_ acquisition delays of 500 ms, 5.4 ms and 2.7 ms respectively. Considering only the relaxation dependent part, peak intensities are modulated by:

1 $${I}^{COCA}=\left[\left(1-{e}^{\left(-{R}_{1}^{{C}{\prime}}(RD+{t}_{2})\right)}\right)\right]\left[{e}^{\left(-{R}_{2}^{{C}{\prime}}2\Delta \right)}{e}^{\left(-{R}_{2}^{{C}^{\alpha }}{t}_{2}\right)}\right]$$

2 $${I}^{CACO}=\left[\left(1-{e}^{\left(-{R}_{1}^{{C}^{\alpha }}(RD+{t}_{2})\right)}\right)\right]\left[{e}^{\left(-{R}_{2}^{{C}^{\alpha }}2\Delta \right)}{e}^{\left(-{R}_{2}^{{C}{\prime}}{t}_{2}\right)}\right]$$

Two parameters contribute to the asymmetry of peak intensities: (i) The different values of indirect and direct acquisition periods: in a COCA-AP experiment, the observable signals relax with R_2_^C’^ during constant time 2∆ and R_2_^Cα^ during t_2_ while the opposite occurs in the CACO-AP experiment; (ii) The choice of the recycle delay: the amount of initial magnetization depends on the progressive saturation of the excited nucleus, this will affect CACO and COCA spectra at different extents.

In order to discuss how the signal intensity in the two experiments is modulated by the geometry of the ligands, we maintained a 5.4 ms 2∆ constant time for the indirect dimension and measured the experimental intensity ratios, I^CACO^/I^COCA^, at different acquisition times t_2,_ as reported in Fig. 6B. When t_2_ is only 10 ms, the four cysteines have a similar behaviour, with CACO experiment being about 40% more sensitive than COCA. The increase of the evolution period t_2_ affects the I^CACO^/I^COCA^ ratio at different extents: for Cys 22, Cys 25 and Cys 34, the ratio increases at longer t_2_ values while an opposite trend is observed for Cys 47. According to Eqs. (1, 2) and keeping constant the recycle delay, the slopes experimentally obtained depend on the difference between R_2_^Cα^ and R_2_^C’^: Cys 22, Cys 25 and Cys 34 have a positive value of ∆R_2_^Cα−C’^, while for Cys 47 the ∆R_2_^Cα−C’^ is negligible or negative. There is a correlation between this finding and the metal-to-carbon distances observed in the NMR structure of PioC (Trindade et al. 2021c): the orientation of Cys 47 is such that C’ and C^α^ are at similar distances from the closest iron ion and in particular the C’ atom is only 4.1 Å from the iron ion. For Cys 22, 25 and 34 the topologies are different, with significantly larger Fe–C’ distances, ≥ 4.9 Å (Fig. 6C). We may speculate that, in the case of Cys 47, both C^α^ and C’ relaxation rates are dominated by the paramagnetic contributions (Querci et al. 2023), and additional contributions to relaxation may result in negative values of ∆R_2_^Cα−C’^. In the other cases, C’ is too far from the Fe ion electron-nucleus dipolar relaxation significantly affect only the C^α^ nuclei. The consequence is that the C’_y_C^α^_z_ coherence, acquired during t_2_ in a CACO experiment, relaxes much slower than the C^α^_y_C’_z_. CACO is therefore the best experiment to observe the C^α^/C’ connectivities of Cys 22, Cys 25 and Cys 34 whereas for Cys 47 the two experiments have the same sensitivity suggesting that, as a general strategy in paramagnetic systems, COCA should be used as a complementary experiment in order to observe C^α^/C’ peaks that might escape detection in CACO.

## Conclusions

A complete, multinuclear NMR assignment provides unique site specific information which are essential to study conformational changes and intermolecular interactions. In metalloproteins, the development of experimental approaches to recover the NMR information in the proximity of a paramagnetic center represents a relevant topic, rejuvenated also in the last years by the extensive use of paramagnetic probes to obtain structural information from paramagnetic NMR (Su and Chen 2019; Dasgupta et al. 2021; Parigi et al. 2023; Ravera et al. 2022; Clore and Iwahara 2009; Clore 2015; Ott et al. 2021; Abelein et al. 2022; Zambelli et al. 2023).

We have analyzed here the performances of HCACO, CACO and COCA, three well known experiments, part of a very successful battery of ^13^C detected experiments (Felli and Pierattelli 2022). We have discussed the relevant modifications needed to adapt these experiments to study site-specific, fast relaxing systems. We also provided examples on how these experiments contribute to improve the available assignments and, therefore, to map the proximity of a metal center. Different coherence transfer pathways call into the scene specific relaxation mechanisms, this should be taken into account for the selection of the experiments to be performed. Apparently redundant experiments, such as CACO and COCA, do contain complementary information which can eventually be converted into structural information.

In the case of PioC, we have corrected the assignment of cluster bound cysteine residues and shown that, even when extended assignment and NMR structure are available, these experiments provide additional information and structural constraints. In the case of CISD3, the optimization of HCACO to paramagnetic system provides a 30% increase of the observed C^α^/C’ signals, a further similar improvement is obtained when the ^1^H start experiment is replaced by the ^13^C start CACO experiment. HCACO and CACO-AP are complementary and reduce the blind sphere around the [Fe_2_S_2_]^+^ cluster from ca 9 to 6 Å.

These experiments are important in the frame of non-systematic sequence-specific assignment strategies: within tailored NMR experiments, the identification of a signal is related to the distance of these spins from the paramagnetic center. This is applicable in all paramagnetic metalloproteins; indeed the only relevant variable is the range of metal to proton distance where conventional NMR experiments do not work properly and complementary experiments must be designed. The information obtained are important to study the interaction of paramagnetic metalloproteins with small ligand molecules (Zuo et al. 2023) and protein partners, particularly for CISD3 and its homologues (Ferecatu et al. 2014; Karmi et al. 2022).

## Materials and methods

### Cisd3 and PioC protein expression and purification

PioC and CISD3 (Ala 37–Leu 127) genes were expressed and purified, as previously reported (Grifagni et al. 2023; Trindade et al. 2021c, 2020). Uniformly ^15^N, ^13^C labelled PioC and CISD3 were expressed in the M9 minimal media with the addition of 1.2 g/L of ammonium sulfate (^15^NH_4_, 99%) and 3.0 g/L of [U–^13^C_6_] D-glucose.

All the purification steps were performed in an anaerobic glovebox, as previously done for other FeS proteins (Maione et al. 2020).

### NMR experiments

All NMR spectra were performed on a Bruker Avance Neo spectrometer operating at a field of 16.4 Tesla and on a Bruker Ascend Neo spectrometer operating at 28.2 T. Both spectrometers were equipped with a TXO probe head optimised for ^13^C carbon direct detection. Experiments were collected at 298 K.

### ^*13*^*C-HCACO and CACO-AP acquired on CISD3 protein*

The standard HCACO (Duma et al. 2003; Bermel et al. 2005b) used a recycle delay of 1 s, acquisition time t_2_ of 92 ms and a H^α^-to-C^α^ INEPT transfer period of 3.6 ms. A 1024 × 660 data point matrix was acquired, with 32 transients for each increment and a spectral width of 31 ppm and 44 ppm for direct (F_2_) and indirect (F_1_) dimensions. For paramagnetic optimized HCACO, the experiment was recorded shortening the H^α^– to –C^α^ INEPT transfer period to 1.4 ms. The acquisition time of the direct dimension, t_2_, was shortened to 46 ms and a recycle delay of 400 ms was used together with 1024 scans. A data matrix of 512 × 50 point was collected, with a spectral width of 31 ppm and 44 ppm for F_2_ and F_1_ dimensions. The CACO-AP (Bertini et al. 2005a) was recorded with 300 ms of recycle delay and with a acquisition time of 24.5 ms. The C^α^-to-C’ INEPT transfer delay was shortened to 5.4 ms instead of 9 ms. The collected data matrix was 512 × 70 with 1024 scans for each increment, with a spectral width of 59 ppm and 60 ppm for F_2_ and F_1_ dimensions. C^α^ carrier was set to 46 ppm for the standard HCACO experiment and to 53.2 ppm for the paramagnetic optimized HCACO and CACO-AP. All the experiments were recorded using waltz65 and garp4 decoupling scheme for ^1^H and ^15^N decoupling, respectively. Smoothed square shape for all gradients was used. Q5 and Q3-shaped pulses, with a duration of 300 μs and 231 μs respectively (Emsley and Bodenhausen 1992), were used for ^13^C band-selective π/2 and π flip angle pulses. Except for HCACO experiments, acquired with the IPAP scheme for virtual decoupling of C^α^_z_C’_y_ component of the magnetization, CACO-AP was processed as pseudo-absorbed signals in the direct dimension, as reported elsewhere (Bertini et al. 2005a). Before Fourier transformation, a square sine weighting function, shifted by 90°, was applied in both dimensions to all the spectra. The evaluation of signal intensity was performed considering the volume of each cross-peak in the spectra (Mori et al. 2008), obtained through an integral operation using Computed-Automated NMR Assignment (CARA) software (Keller 2004).

### ^*13*^*C-HCACO, CACO-AP and COCA-AP acquired on PioC protein*

Optimized HCACO-AP was acquired with recycle delay of 250 ms, acquisition time of 46 ms and an H^α^-to-C^α^ INEPT transfer delay of 1.4 ms. The total data matrix for the experiment was 512 × 128 point, with 1024 transients for each increment and a spectral width of 31 and 80 ppm for F_2_ and F_1_ dimensions. A further optimized HCACO-AP was acquired with a data matrix of 512 × 100, with 7168 scans for each increment, and a H^α^-to-C^α^ INEPT transfer delay of 1 ms. The recycle delay and acquisition time for the experiment were 250 ms and 46 ms, respectively. The spectral width for F_2_ and F_1_ dimensions were 21 ppm and 64 ppm, respectively. CACO-AP and COCA-AP experiments were recorded both with recycle delay, acquisition time and C^α^-to-C’ INEPT transfer delay of 500 ms, 47 ms and 5.4 ms, respectively. A data matrix of 2048 × 120 points, with 128 transients for each increment, was collected and a spectral width of 123 ppm and 64 ppm for direct and indirect dimensions has been used. The H(CA)CO-AP experiment was acquired with recycle delay, acquisition time and H^α^-to-C^α^ INEPT transfer delay of 250 ms, 46 ms and 1.4 ms. A data matrix of 512 × 220 point was collected, with 512 transients for each increment. The spectral width for direct and indirect dimension were 31 ppm, 64 ppm and 11 ppm, respectively. Shaped pulses, ^1^H and ^15^N decoupling, processing parameters were as described above. The optimization of HCACO experiment was monitored at 28.2 T, using a series of experiments, recorded with the same acquisition parameters. Experiments were recorded with 128 ns, 1 s recycle delay, 9.6 ms and 50 ms as t_1_ and t_2_ acquisition dimensions. Each experiment was 5 h. Throughout this series of experiments, ^15^N CPD was achieved using a p5m4sp180 decoupling scheme. An 8 ms, smoothed adiabatic chirp pulse with 8.4 kHz sweep width was used.

### Supplementary Information

Below is the link to the electronic supplementary material.

Supplementary file1 (DOCX 34 KB)
