# Supplementary material for: Paramagnetic NMR to study iron sulfur proteins: 13C detected experiments illuminate the vicinity of the metal center
Source: J Biomol NMR. 2023 Oct 18;77(5-6):247–59. doi: 10.1007/s10858-023-00425-4 (PMC10687126; doi:10.1007/s10858-023-00425-4)
Supplement: Supplementary file 1 — Supplementary file1 (DOCX 34 KB) [file 10858_2023_425_MOESM1_ESM.docx]

Paramagnetic NMR to Study Iron Sulfur Proteins: ^13^C detected experiments illuminate the vicinity of the metal center

*Leonardo Querci^a^, Deborah Grifagni^a^, Ines B Trindade^b^, Jose Malanho Silva^a^, Ricardo O Louro^b^, Francesca Cantini^a^, Mario Piccioli^a*^*

**SUPPORTING INFORMATION**

**Table S1**: H^α^-Fe, C^α^-Fe and C’-Fe distances (from the closer iron ion) measured from the crystallographic structure of Cisd3 (PDB ID: 6AVJ). The two CDGSH domains span from Leu 43 to Thr 80 and from Gly 81 to Val 118. Columns 3 and 9 indicate the closest cluster considered for the reported distances. Cluster 1 is coordinated by Cys 60, Cys 62, Cys 71 and His 75. Cluster 2 is coordinated by Cys 98, Cys100, Cys 109 and His 113. Orange and blue color labelling highlight residues within 10 Å from the cluster. Distances highlighted in green were used to propose the assignment of the corresponding C^α^/C’ connectivities in the HCACO and CACO-AP spectra. Metal-to-nuclei distances for residues Met 35, Gly 36 and all the Histidine residues are missing because they were not present in the crystallographic structure of Cisd3 protein (PDB ID: 6AVJ).

| Number | Residue | Cluster | H^α^-Fe distance | C^α^-Fe distance | C'-Fe distance | Number | Residue | Cluster | H^α^-Fe distance | C^α^-Fe distance | C'-Fe distance |
| --- | --- | --- | --- | --- | --- | --- | --- | --- | --- | --- | --- |
| 35 | Met | Cluster 1 |  |  |  |  |  |  |  |  |  |
| 36 | Gly | Cluster 1 |  |  |  |  |  |  |  |  |  |
| 37 | Ala | Cluster 1 | 11.9 | 11.0 | 10.7 |  |  |  |  |  |  |
| 38 | Arg | Cluster 1 | 11.5 | 11.2 | 9.9 |  |  |  |  |  |  |
| 39 | Ser | Cluster 1 | 7.7 | 8.5 | 9.2 |  |  |  |  |  |  |
| 40 | Val | Cluster 1 | 10.3 | 9.4 | 9.3 |  |  |  |  |  |  |
| 41 | Val | Cluster 1 | 7.6 | 8.4 | 8.9 |  |  |  |  |  |  |
| 42 | Ala | Cluster 2 | 10.1 | 9.1 | 9.0 |  |  |  |  |  |  |
| 43 | Leu | Cluster 2 | 8.9 | 8.1 | 7.0 | 81 | Gly | Cluster 1 | 10.3 | 10.0 | 9.1 |
| 44 | Lys | Cluster 2 | 6.2 | 6.9 | 6.3 | 82 | Leu | Cluster 1 | 7.9 | 7.3 | 6.2 |
| 45 | Thr | Cluster 2 | 5.2 | 5.9 | 5.6 | 83 | Ser | Cluster 1 | 4.6 | 5.4 | 5.2 |
| 46 | Pro | Cluster 2 | 4.8 | 5.0 | 6.5 | 84 | Pro | Cluster 1 | 4.6 | 4.9 | 6.4 |
| 47 | Ile | Cluster 2 | 8.9 | 8.5 | 8.9 | 85 | Leu | Cluster 1 | 8.8 | 8.4 | 8.9 |
| 48 | Lys | Cluster 2 | 10.0 | 10.5 | 11.7 | 86 | Lys | Cluster 1 | 9.6 | 10.1 | 11.5 |
| 49 | Val | Cluster 2 | 13.8 | 13.2 | 14.1 | 87 | Phe | Cluster 1 | 13.8 | 13.3 | 14.0 |
| 50 | Glu | Cluster 2 | 16.1 | 16.4 | 16.8 | 88 | Lys | Cluster 1 | 15.8 | 16.2 | 17.1 |
| 51 | Leu | Cluster 2 | 17.1 | 17.0 | 18.5 | 89 | Ala | Cluster 1 | 18.0 | 18.2 | 19.6 |
| 52 | Val | Cluster 2 | 21.1 | 20.4 | 20.4 | 90 | Gln | Cluster 1 | 21.8 | 21.0 | 21.4 |
| 53 | Ala | Cluster 2 | 21.8 | 21.9 | 21.6 | 91 | Glu | Cluster 2 | 21.8 | 20.8 | 20.1 |
| 54 | Gly | Cluster 1 | 20.9 | 20.8 | 19.5 | 92 | Thr | Cluster 2 | 19.9 | 20.5 | 19.6 |
| 55 | Lys | Cluster 1 | 17.8 | 17.6 | 16.4 | 93 | Arg | Cluster 2 | 18.1 | 17.6 | 16.4 |
| 56 | Thr | Cluster 1 | 14.6 | 14.3 | 13.1 | 94 | Met | Cluster 2 | 14.7 | 14.6 | 13.4 |
| 57 | Tyr | Cluster 1 | 11.1 | 11.3 | 10.1 | 95 | Val | Cluster 2 | 11.2 | 11.2 | 10.1 |
| 58 | Arg | Cluster 1 | 8.0 | 7.6 | 7.0 | 96 | Ala | Cluster 2 | 8.1 | 7.7 | 7.0 |
| 59 | Trp | Cluster 1 | 7.6 | 7.2 | 5.8 | 97 | Leu | Cluster 2 | 7.1 | 7.1 | 5.9 |
| 60 | Cys | Cluster 1 | 3.3 | 3.8 | 4.2 | 98 | Cys | Cluster 2 | 3.3 | 4.8 | 5.7 |
| 61 | Val | Cluster 1 | 5.7 | 5.0 | 4.7 | 99 | Thr | Cluster 2 | 5.6 | 4.9 | 4.6 |
| 62 | Cys | Cluster 1 | 5.0 | 4.1 | 4.6 | 100 | Cys | Cluster 2 | 4.9 | 4.0 | 4.4 |
| 63 | Gly | Cluster 1 | 6.1 | 5.2 | 5.2 | 101 | Lys | Cluster 2 | 4.9 | 5.0 | 5.2 |
| 64 | Arg | Cluster 1 | 5.9 | 5.0 | 5.0 | 102 | Ala | Cluster 2 | 6.0 | 5.2 | 5.0 |
| 65 | Ser | Cluster 1 | 4.9 | 4.7 | 6.2 | 103 | Thr | Cluster 2 | 4.6 | 4.7 | 6.3 |
| 66 | Lys | Cluster 1 | 8.7 | 8.5 | 9.0 | 104 | Gln | Cluster 2 | 8.7 | 8.4 | 9.0 |
| 67 | Lys | Cluster 1 | 9.9 | 9.0 | 8.6 | 105 | Arg | Cluster 2 | 10.0 | 9.1 | 8.6 |
| 68 | Gln | Cluster 1 | 7.4 | 8.3 | 9.4 | 106 | Pro | Cluster 2 | 7.5 | 8.4 | 9.5 |
| 69 | Pro | Cluster 1 | 8.1 | 8.4 | 7.4 | 107 | Pro | Cluster 2 | 8.2 | 8.6 | 7.5 |
| 70 | Phe | Cluster 1 | 6.9 | 7.0 | 5.9 | 108 | Tyr | Cluster 2 | 7.3 | 7.3 | 5.9 |
| 71 | Cys | Cluster 1 | 3.3 | 3.8 | 4.5 | 109 | Cys | Cluster 2 | 3.2 | 3.8 | 4.4 |
| 72 | Asp | Cluster 1 | 6.0 | 5.4 | 5.1 | 110 | Asp | Cluster 2 | 5.8 | 5.3 | 5.0 |
| 73 | Gly | Cluster 1 | 4.1 | 4.4 | 4.6 | 111 | Gly | Cluster 2 | 4.0 | 4.4 | 4.7 |
| 74 | Ser | Cluster 1 | 6.1 | 5.2 | 5.0 | 112 | Thr | Cluster 2 | 6.2 | 5.3 | 5.1 |
| 75 | His | Cluster 1 |  |  |  | 113 | His | Cluster 2 |  |  |  |
| 76 | Phe | Cluster 1 | 7.0 | 6.6 | 7.8 | 114 | Arg | Cluster 2 | 6.9 | 6.8 | 8.0 |
| 77 | Phe | Cluster 1 | 9.5 | 8.9 | 9.5 | 115 | Ser | Cluster 2 | 9.9 | 9.2 | 9.9 |
| 78 | Gln | Cluster 1 | 10.5 | 9.6 | 9.9 | 116 | Glu | Cluster 2 | 12.0 | 12.2 | 12.0 |
| 79 | Arg | Cluster 1 | 9.8 | 10.2 | 10.1 | 117 | Arg | Cluster 2 | 12.3 | 11.5 | 10.4 |
| 80 | Thr | Cluster 1 | 9.5 | 9.0 | 9.5 | 118 | Val | Cluster 2 | 8.4 | 8.3 | 9.0 |
|  |  |  |  |  |  | 119 | Gln | Cluster 2 | 9.7 | 10.3 | 11.5 |
|  |  |  |  |  |  | 120 | Lys | Cluster 2 | 13.8 | 13.2 | 13.1 |
|  |  |  |  |  |  | 121 | Ala | Cluster 2 | 12.6 | 11.9 | 12.3 |
|  |  |  |  |  |  | 122 | Glu | Cluster 2 | 13.6 | 13.5 | 12.7 |
|  |  |  |  |  |  | 123 | Val | Cluster 2 | 11.2 | 12.1 | 13.0 |
|  |  |  |  |  |  | 124 | Gly | Cluster 2 | 13.5 | 12.0 | 12.3 |
|  |  |  |  |  |  | 125 | Ser | Cluster 2 | 14.7 | 13.7 | 12.9 |
|  |  |  |  |  |  | 126 | Pro | Cluster 2 | 12.0 | 12.7 | 12.3 |
|  |  |  |  |  |  | 127 | Leu | Cluster 2 | 11.0 | 11.1 | 12.3 |
